# Natural history of human papillomavirus and vaccinations in men: A literature review

**DOI:** 10.1002/hsr2.118

**Published:** 2019-03-12

**Authors:** Benjamin J. Lieblong, Brooke E. E. Montgomery, L. Joseph Su, Mayumi Nakagawa

**Affiliations:** ^1^ College of Medicine, Department of Pathology University of Arkansas for Medical Sciences Little Rock Arkansas USA; ^2^ Faye W. Boozman College of Public Health, Department of Health Behavior and Health Education University of Arkansas for Medical Sciences Little Rock Arkansas USA; ^3^ Faye W. Boozman College of Public Health, Department of Epidemiology University of Arkansas for Medical Sciences Little Rock Arkansas USA

**Keywords:** human papillomavirus, men, prophylactic vaccination, therapeutic vaccination

## Abstract

**Background and aims:**

Infection with high‐risk (HR) genotypes of the human papillomavirus (HPV) is necessary for and causative of almost all cervical cancers and their precursor condition, cervical intraepithelial neoplasia. These conditions have been sharply reduced by cervical cytology screening, and a further decrease is expected because of the recent introduction of prophylactic HPV vaccinations. While significant attention has been given to gynecologic HPV disease, men can be affected by HPV‐related cancers of the anus, penis, and oropharynx. This literature review aims to address disparities in HPV‐related disease in men, and certain HR male subpopulations, compared with women.

**Discussion:**

Overall, immunocompetent men are far less likely than women to develop anogenital HPV‐related cancers, despite harboring HR HPV infections at anogenital sites. On the other hand, men who have sex with men and men living with human immunodeficiency virus infection are at considerably higher risk of HPV‐related disease. Historic rates of prophylactic HPV vaccination in males have trailed those of females due to numerous multilevel factors, although, in recent years, this sex gap in vaccination coverage has been closing. In the absence of routine HPV screening in males, therapeutic vaccinations have emerged as a potential treatment modality for preinvasive neoplasia and are in various phases of clinical testing.

**Conclusion:**

Successful reductions in HPV disease morbidity at the population level must acknowledge and target HPV infections in men.

## 1 INTRODUCTION

While cancer can have numerous etiologies, including environmental triggers, genetic predispositions, and carcinogenic agents, one of the more intriguing causes of cancer is infection with oncogenic microorganisms. In fact, infections predominantly with viruses and some bacteria are responsible for approximately 20% of worldwide cancer cases.^1^ One such virus, the human papillomavirus (HPV), comprises a family of over 200 genotypes, which can be loosely categorized based on oncogenicity into low‐risk (LR) and high‐risk (HR) genotypes. LR genotypes, such as 6 and 11, among others, preferentially infect cutaneous sites to cause skin warts and condyloma acuminata.^2^ HR genotypes, such as 16, 18, 31, 33, 45, 52, and 58, among others, preferentially infect mucosal sites and are associated with the development of certain vaginal, vulvar, cervical, penile, anal, and oropharyngeal cancers.^3^ Although HPV is efficiently transmitted via sexual contact, HPV is an epithelial virus that can spread through skin‐to‐skin contact not necessarily requiring insertive intercourse.^4^ While most cervical HPV infections are cleared without incident, some may progress to cervical cancer, the fourth most common cancer worldwide in women.^5^ The overwhelming majority of cervical cancer cases are linked to HR HPV infections.^6^, ^7^ The implementation of Papanicolaou (Pap) screening, alone or in combination with HPV deoxyribonucleic acid (DNA) testing, has precipitously decreased the incidence of cervical cancer.^8^ Prophylactic HPV vaccination stands to sharply decrease cervical cancer incidence rates, which is suggested by the decrease in cervical precancerous lesions after large‐scale vaccination programs.^9^

While large‐scale screening and vaccination programs have begun to address HPV morbidities in women, men are not spared from potentially cancerous HPV infection. Men can succumb to complications stemming from LR HPV infection (nominally, genital warts) and HR HPV infection (such as penile and anal cancers), although the course of naturally acquired HPV infections in men is less studied than that of women with cervical infections.^10^ However, in contrast to women, men are far less likely to develop HPV‐related cancers, despite harboring persistent infection with HR HPV and having lower HPV seroprevalence compared with women.^11^, ^12^ The combination of these facets of HPV infection in men (HIM) have led to the reputation of men as a viral reservoir.^13^ Additionally, a woman's risk of developing cervical cancer has been shown to be related to the sexual behavior patterns of males with HPV.^14^ Thus, here, we hope to shed light on the role of males as a viral reservoir and the critical role that prophylactic and/or therapeutic vaccination in males can play in reducing HR HPV–related morbidity in both sexes.

## 2 GENITAL, OROPHARYNGEAL, AND ANAL HPV INFECTION IN MEN

In comparison with women, men are much less likely to incur genital HPV complications.^15^ Although women may presently carry a higher genital HPV disease burden, current epidemiologic trends show decreasing incidence of cervical dysplasia and cancer in developed nations, largely due to routine Pap screening but aided in part by prophylactic vaccination.^5^, ^16^, ^17^ In contrast to routine Pap testing in conjunction with HPV DNA testing in women, no such screening tests are currently available for men.^18^ In a research setting, numerous groups have reported HPV DNA testing at numerous genital sites, including the glans penis, coronal sulcus, urethra, scrotum, and perineum.^19^, ^20^, ^21^, ^22^, ^23^ The HIM cohort study of 1159 men residing in Brazil, Mexico, and the United States estimated the prevalence of any HPV, oncogenic HPV, and nononcogenic genotypes at 50%, 30%, and 38%, respectively, using samples from the coronal sulcus, glans penis, penile shaft, and scrotum. The same study reported a median time to clearance of oncogenic HPV genotypes of 7.2 months and 12.2 months for HPV 16 specifically.^24^ While penile HPV prevalence may vary based on sampling/screening techniques, these persistent penile HPV infections pose a risk of disease progression. Although cancers of the penis are rare, penile oncogenesis arises due to persistent infection with HR genotypes of HPV, and first present as penile intraepithelial neoplasia (PIN).^25^, ^26^ HR genotypes of HPV, including HPV 16, are associated with the development of PIN in younger men.^27^ Initial low‐grade PIN lesions typically resolve within 2 years; however, similar to CIN, a small minority of cases may progress to high‐grade PIN lesions^28^, ^29^ that could result in invasive cancer. In fact, a meta‐analysis of 1266 invasive penile squamous cell carcinoma cases from 30 studies demonstrated an HPV prevalence of 47.9%, with an HPV 16 and/or HPV 18 prevalence of 36.7%.^25^

Penile cancer in the United States has an incidence rate of approximately one per 100 000, while the incidence in developing nations can be much higher.^11^ Uganda, for example, has an estimated age‐specific incidence of penile cancer^30^ of 4.4 per 100 000. An exact mechanism to explain this incidence difference has yet to be described, but several theories exist. Penile HPV infection and PIN are known to occur at higher rates in immunocompromised patients, such as patients infected with human immunodeficiency virus (HIV).^31^, ^32^ While Ugandan adult HIV seroprevalence has dropped from approximately 14% in 1990, at the peak of the HIV epidemic, to approximately 5% in 2007, adult HIV infection and its sequelae remain a major public health concern.^33^ An interesting trend discussed in great detail elsewhere^34^, ^35^ is the reduced risk of penile HPV infection in circumcised males compared with uncircumcised males. While a biological mechanism has yet to be described, numerous studies have shown a lower incidence of overt penile cancer, PIN lesions, and penile HPV DNA detection in circumcised men compared with uncircumcised men.^36^, ^37^, ^38^, ^39^ Examining both the effects of HIV serostatus and circumcision status, one group has shown a dually protective effect of circumcision and HIV‐negative serostatus in sub‐Saharan African men.^40^, ^41^ Identifying factors influencing or exacerbating male genital HPV infection complications is an area of active research and continues to provide etiologic bases for understanding the natural history of male penile HPV infection.

A facet of HPV infection that has gained greater appreciation in recent years is the link between HPV infection and head/neck malignancies, most notably, oropharyngeal squamous cell carcinomas (OSCCs). Cancers of the head and neck can be divided into two distinct forms, which vary in epidemiology, etiology, and treatments.^42^ Traditionally, oropharyngeal cancers have been linked primarily to tobacco use, but also to alcohol, poor oral hygiene, and certain genetic predispositions.^43^ These non‐HPV–related head and neck cancers have been declining in recent years concomitantly with declines in tobacco use.^44^ However, incidence of HPV‐associated oropharyngeal cancer appears to be rising.^45^ It is now appreciated that some 40% to 80% of oropharyngeal cancers in the United States are caused by HPV, 90% of which have been linked^46^ to HPV 16. The natural history of oral HPV remains less clear than that of anogenital HPV infection (cervical, in particular), but several studies have linked oral HPV infection (HR HPV, in particular) with changes in sexual behavior, such as younger age of first oral sex and increasing lifetime numbers of sexual partners, particularly among younger adults.^47^, ^48^, ^49^, ^50^ The natural decline of non‐HPV–related OSCC (as a result of less tobacco use among younger individuals) coupled with the increase of HPV‐related OSCC (as a result of changing sexual norms among younger individuals) has been hypothesized to accentuate the outcomes of HPV‐related OSCC in the coming years.^46^

Of particular relevance to this review, oral HPV infection and associated OSCC are far more prevalent in males compared with females. Of 5501 individuals aged 14 to 69 who participated in the National Health and Nutrition Examination Survey (NHANES) in 2009 to 2010, overall prevalence of oral HPV (of any genotype) was approximately 7%. Prevalence of oral HPV infection followed a bimodal distribution in both sexes, with peaks at age 30 to 34 and 60 to 61 years. However, oral HPV infection prevalence was approximately threefold higher in men than women, and the prevalence of HPV 16 was over fivefold higher in men.^51^ Analyzing the same year cohort of NHANES participants, another group reported that male sex (i.e., being male) and oral sexual behavior are both associated with oral HPV 16 infection, even when controlling for age‐cohort and race,^47^ suggesting a primary role of sexual behavior with oral HIM. Moreover, these studies have shown greater prevalence of *infection* in men, and HPV‐associated OSCC has been shown to be more common in men, both in the United States and other developed nations.^52^, ^53^, ^54^ HPV infections and associated cancers unfortunately suffer from a public misperception as a health issue unique to women^55^; however, current HPV‐related cancer incidence trends underscore the opposite. In the United States, the incidence of HPV‐related OSCC in men has exceeded the incidence of cervical cancer, which is on the decline.^56^ In light of this increased prevalence of both HPV infection and overt OSCC in males, further attention must be given to male patients in both prophylaxis and screening.

The causal link between cervical HPV infection and associated cervical dysplasia and carcinoma has a similar parallel in anal HPV infection, anal dysplasia, and its progression to invasive carcinoma. In both men and women, the incidence of anal cancer has increased by approximately 2% per year since^57^ 1970. Interestingly, women with a history of previous cervical intraepithelial lesions have been shown to be at increased risk of HPV‐related anal disease.^58^, ^59^ A meta‐analysis of 29 studies on anal lesions concluded that over 80% of anal dysplasia and carcinoma cases are associated with HPV infection.^60^ Of 102 male patients and 146 female anal cancer patients in the Seattle, Washington area, 86.3% and 89.0% of tumor samples were found to be positive for HPV DNA, respectively.^61^ Thus, anal HPV infection seems to be strongly associated with the development of invasive anal carcinoma.

HPV infections are not systemic but rather localized to the anogenital mucosal sites of viral exposure. As such, anal HPV cases are typically associated with receptive anal sex, a sexual practice common—but not unique—to men who have sex with men (MSM). Numerous studies have shown that anal HPV prevalence (defined as detection of DNA from one or more HPV genotypes from anal swab samples) trends higher among MSM, HIV‐positive, and HIV‐positive MSM populations (Table 1). In the previously mentioned study of Seattle, Washington, anal cancer patients by Daling and colleagues, 48 of 102 (47%) male patients self‐identified as not exclusively heterosexual. In anal tumors from this MSM population, 97.7% were found to be positive for HPV DNA.^61^ A cross‐sectional study of 401 HIV‐positive MSM revealed abnormal anal cytology in 67% of study participants, histologic abnormalities in 68%, and HPV DNA detection (of any genotype) in 93%.^73^ Thus, receptive anal intercourse is recognized as an established risk factor for anal HPV infection, anal intraepithelial neoplasia (AIN), and anal carcinoma.

**Table 1:** Anal HPV prevalence of male patients, stratified by HIV serostatus and sexual activity

Study Population			Types Detected	Anal HPV Prevalence (%)				Country	Reference
HIV Status and Sexual Activity	Size (n)	Specification		HPV 16	Any HR	Any LR	Any HPV		
HIV^−^ MSW	85	5 or more sexual partners in the 6 mo prior to study entry	6/11, 16, 18, 33	1.2	1.2	0	1.2	Netherlands	van Doornum et al^62^
	50	Stable sexual partners of women positive for HPV by HC	HC	NR	4.0	2.0	8.0	Brazil	Nicolau et al^63^
	222	MSW with no history of oral or anal sex with men	LA	0.9	5.4	9.0	16.6	USA	Nyitray et al^64^
	902	MSW in the HIM cohort	LA	3.2	7.0	7.0	12.0	USA, Brazil, Mexico	Nyitray et al^65^
	1305	MSW with only female partners in the last 6 mo and <3 lifetime male sex partners	LA	2.2	6.8	5.4	12.2	USA, Brazil, Mexico	Nyitray et al^66^
HIV^+^ MSW	50	IVDU patients of an HIV outpatient clinic	6, 11, 16, 18, 26, 31‐33, 35, 39, 40, 45, 51‐56, 58, 59, 61, 66, 68‐70, 73, AE2, *Pap* 155, *Pap* 291, 2/13/34/42/57/62/64/67/72/W13B	NR	44.0	44.0^a^	46.0	USA	Piketty et al^67^
	68	Patients of the HIV outpatient clinic of a university‐affiliated teaching hospital	6, 11, 16, 18, 26, 31‐35, 39, 40, 42‐45, 51‐59, 61, 62, 66, 68‐70, 73	5.8	25.0	NR	51.5	Korea	Lee et al^68^
HIV^−^ MSM	200	MSM and MSMW	6, 11, 16, 18, 26, 31‐33, 35, 39, 40, 45, 51‐56, 58, 59, 61, 66, 68‐70, 73, AE2, *Pap* 155, *Pap* 291, 2/13/34/42/57/62/64/67/72/W13B	19.0	29.0	16.0	61.0	USA	Palefsky et al^69^
	93	Sydney, Australia men with at least one male sex partner in the previous 5 y	LA	26.9	73.1	87.1	93.5	Australia	Vajdic et al^70^
	116	Self‐reported history of receptive anal intercourse	LA	NR	51.7	64.7	75.0	Slovenia	Milošević et al^71^
	1218	History of anal intercourse with ≥1 man during the preceding year	6, 11, 16, 18, 26, 31‐33, 35, 39, 40, 45, 51‐56, 58, 59, 61, 66, 68‐70, 73, AE2, *Pap* 155, *Pap* 291, 2/13/34/42/57/62/64/67/72/W13B	12.0	22.0	22.0	57.0	USA	Chin‐Hong et al^72^
	176	MSM and MSMW	LA	6.3	27.3	19.9	47.2	USA, Brazil, Mexico	Nyitray et al^66^
HIV^+^ MSM	400	Patients of tertiary care hospital HIV clinics, history of receptive anal intercourse	HC + LA	38.0	88.0	NR	93.0	Canada	Salit et al^73^
	20	Self‐reported history of receptive anal intercourse	LA	NR	85.0	95.0	95.0	Slovenia	Milošević et al^71^
	36	Sydney, Australia men with at least one male sex partner in the previous 5 y	LA	36.1	94.4	88.9	97.2	Australia	Vajdic et al^70^
	289	MSM and MSMW	6, 11, 16, 18, 26, 31‐33, 35, 39, 40, 45, 51‐56, 58, 59, 61, 66, 68‐70, 73, AE2, *Pap* 155, *Pap* 291, 2/13/34/42/57/62/64/67/72/W13B	38.0	80.0	63.0	93.0	USA	Palefsky et al^69^
	67	Non‐IVDU MSM patients of an HIV outpatient clinic	6, 11, 16, 18, 26, 31‐33, 35, 39, 40, 45, 51‐56, 58, 59, 61, 66, 68‐70, 73, AE2, *Pap* 155, *Pap* 291, 2/13/34/42/57/62/64/67/72/W13B	NR	65.0	35.0^a^	85.0	USA	Piketty et al^67^
	133	Patients of the HIV outpatient clinic of a university‐affiliated teaching hospital	6, 11, 16, 18, 26, 31‐35, 39, 40, 42‐45, 51‐59, 61, 62, 66, 68‐70, 73	13.5	47.4	NR	82.7	Korea	Lee et al^68^

Abbreviations: HC, Hybrid Capture (Qiagen, Hilden, Germany); genotypes detected: 16/18/31/33/35/39/45/51/52/56/58/59/68 and 6/11/42/43/44; HIM, HPV infection in men; HPV, human papillomavirus; HR, high risk; IVDU, intravenous drug users; LA, linear array (Roche Molecular Diagnostics, Pleasonton, CA, USA); genotypes detected: 6, 11, 16, 18, 26, 31, 33, 35, 39, 40, 42, 45, 51, 52, 53, 54, 55, 56, 58, 59, 61, 62, 64, 66, 67, 68, 69, 70, 71, 72, 73 (MM9), 81, 82 (MM4), 83 (MM7), 84 (MM8), IS39, and CP6108; LR, low risk; MSM, men who have sex with men; MSMW, men who have sex with men and women; NR, not reported.

a LR HPV positivity in the study by Piketty et al^67^ is reported as LR *only* and therefore may be artificially low due to the mutual exclusivity of this statistic with HR HPV positivity.

While anal HPV has been well characterized in MSM populations, receptive anal intercourse does not appear to be necessary for anal infection. A study comparing 50 HIV‐positive male intravenous drug users with no history of anal intercourse and 67 HIV‐positive MSM showed a 46% and 85% HPV DNA positivity, respectively. This study also reported similar incidences of anal high‐grade squamous intraepithelial lesions (HSIL) in both populations (18%).^67^ Outside of the HIV context, a few groups have shown HPV DNA in the perianal or anal canal regions of men who have sex with women (MSW). A 1994 study of 162 women and 85 MSW at an Amsterdam STI clinic showed an anal HPV prevalence of 2.5% and 1.2%, respectively.^62^ A 2005 study of 50 male sexual partners of HPV‐infected women showed anal HPV prevalence of 8%.^63^ A 2008 study of MSW with no self‐reported lifetime history of sex (oral or anal) with men showed a prevalence of anal HPV of 24.8%. These authors also reported a 34.4% concordance rate between the HPV types detected at anal (anal canal and perianal region) and genital (urethra, glans penis, penile shaft, and scrotum) sites.^64^ A 2010 study from the HPV in men (HIM) cohort identified anal canal HPV prevalence at 12.0%, although this study's MSW population included men who may have had sex with one or two men in their lifetime, but not recently.^65^ A final study from the HIM cohort directly compared MSW (n = 1305) and MSM (n = 176) and showed anal canal HPV prevalence of 12.2% and 47.2%, respectively.^66^ In the MSM population of this study, a younger age and higher numbers of sexual partners were independently associated with detection of HPV in the anal canal. Although anal cytology screening has been performed, its predictive value of oncogenic progression (including a time frame) remains controversial.^11^ The prevalence statistics of the aforementioned studies may vary due to differences in HPV DNA detection techniques or study population heterogeneity, and others report evidence of auto‐innoculation from genital and manual sites.^74^, ^75^ Nonetheless, there is a small but substantive body of evidence that male anal HPV infection can occur in the absence of a history of anal intercourse.

Because the overwhelming majority of HPV infections resolve within 2 years, an important epidemiological aspect of male HPV infection is seroprevalence, which can provide a measure of viral exposure. Serum antibodies may serve as a surrogate marker for ongoing or previous infections, or even lifetime exposure.^76^, ^77^, ^78^, ^79^, ^80^ Anti‐HPV antibodies can persist for years,^81^ with one group showing stable levels of anti‐HPV 16 antibodies for a follow‐up period of 7 to 13 years,^82^ although others suggest that antibody response duration is variable.^83^ While serum anti‐HPV antibodies are not necessarily neutralizing antibodies to aid in infection clearance, they may provide protection against infection by the same HPV types, which has been shown in both patients and a canine model.^84^, ^85^ Although HPV seroconversion data may provide useful surrogate markers of viral exposure, not all who have an HPV infection will seroconvert.^86^ When accounting for this caveat in HPV serobiology, understanding HPV seroprevalence can provide population‐level analytics of viral spread and/or disease progression.

**Figure 1:**
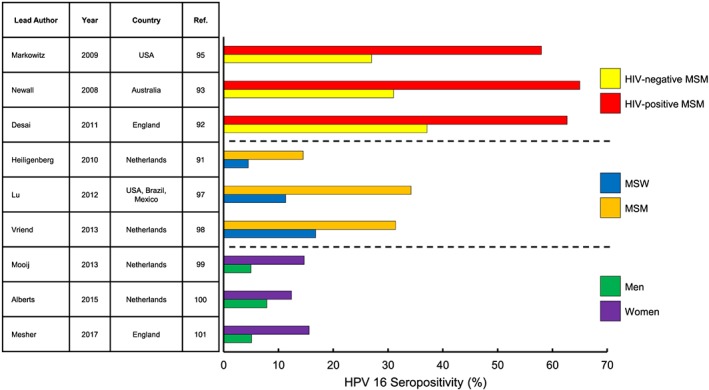
Seroprevalence of HPV 16 in patients of various subpopulations. HPV 16 seroprevalence, defined as positive detection of anti‐HPV 16 antibodies, in patients of various subpopulations. Dashed lines delineate subpopulation comparisons in each cluster of three studies. Reported values are percentage of patients seropositive for HPV 16 in each study. HPV, human papillomavirus; MSM, men who have sex with men; MSW, men who have sex with women

Numerous cross‐sectional and longitudinal studies of HPV antibody responses have shown lower HPV seroprevalence in men compared with women. Women disproportionately produce HPV antibody responses to HPV 16 compared with men.^12^, ^87^, ^88^, ^89^, ^90^, ^91^, ^92^, ^93^ A study examining HPV seroprevalence of Greenlandic patients seeking treatment in a sexually transmitted infection clinic showed anti‐HPV 16 virus‐like particle (VLP) antibodies in 35% of male subjects compared with 75% of female subjects.^87^ The same study examined Danish patients, which had 32% and 56% HPV 16 seropositivity in men and women, respectively.^87^ While other studies may have reported lower overall incidence of seropositivity, a parallel sex gap still exists. For example, an English population‐based sample of males and females aged 10 to 49 years showed HPV 16 seropositivity rates of 5.0% and 14.7% in men and women, respectively.^92^ Indeed, Australia, a country that enjoys some of the highest prophylactic HPV vaccination rates in the world^94^ shows lower overall HPV 16 seroprevalence in men (7.9%) compared with women (12.4%) in a population‐based study.^93^ A study of US males and females aged 14 to 59 years who participated in the NHANES in 2003 to 2004 confirmed higher seroprevalence in women not only for HPV 16 but also for the three remaining genotypes covered by the quadrivalent (4V) Gardasil vaccine.^95^ Data generated in other studies using NHANES data again confirmed the sex gap in HPV 16 seropositivity.^96^ While seroprevalence is lower in the general male population compared with females, certain male patient subpopulations can have much higher seroprevalence (Figure 1). Numerous studies have shown greater than or equal to twofold higher HPV 16 seropositivity in MSM compared with MSW.^91^, ^97^, ^98^ Within MSM populations, HIV serostatus appears strongly associated with HPV seropositivity. Reported HPV 16 seroprevalence in HIV‐negative MSM ranges from 27.0% to 37.1%, compared with 58.0% to 65.0% in HIV‐positive MSM.^99^, ^100^, ^101^ A caveat that must be acknowledged in the analysis of HPV seroprevalence data is that seroconversion lags behind detection of viral DNA at the site of suspected infection, and at times, this can be absent altogether.^102^ An additional caveat is the relationship of anatomic site of infection and likelihood of seroconversion. The higher seropositivity for HPV in women compared with men may be partially explained by observations that HPV infections of mucosal epithelium, such as the cervix, generate stronger antibody responses than infections at cutaneous sites, such as the penile skin.^90^, ^97^, ^98^ Nevertheless, the aforementioned studies collectively suggest that men do not mount as strong an immune response to HPV as women do, which can leave men vulnerable to cyclical reinfection and subsequent HPV transmission to their female sex partners, thereby substantiating the role of men as a viral reservoir.

## 3 PROPHYLACTIC VACCINATION

Second to the widespread adoption of cervical cytology screening, the next public health success in the HPV context was the development of prophylactic HPV vaccines. Three vaccines in total have been approved: 4V Gardasil (Merck), bivalent Cervarix (GlaxoSmithKline), and nonavalent Gardasil 9 (Merck). Although genotype coverage varies by vaccine, each of the approved vaccines consists of synthetically made HPV VLPs, which are generated in vitro using a viral capsid protein L1 epitope.^103^ Quadrivalent Gardasil, the first prophylactic HPV vaccine, confers protection against genotypes 6, 11, 16, and 18 and was approved by the USA Food and Drug Administration (FDA) in 2006 for prophylaxis of CIN and cancer in females ages 9 to 26. Quadrivalent Gardasil was first approved for use in males ages 9 to 26 for prevention of condyloma acuminata in 2009, followed by indications for AIN prevention in both sexes^104^ in 2010. Cervarix confers protection against genotypes 16 and 18 and was FDA approved^105^ in 2009; however, its use in the United States has been blunted due to decreased market demand secondary to greater genotype coverage in other vaccines.^106^ The most recently developed prophylactic HPV vaccine, Gardasil 9, confers protection against all genotypes covered by 4V Gardasil and five additional HR genotypes: 31, 33, 45, 52, and 58. The indication of Gardasil 9 at its release in December 2014 included prevention of AIN and condyloma acuminata in males ages 9 to 15. Approximately 1 year later, the approved vaccination age for males was increased to ages 9 to 26, which matches the approved ages for females.^107^ The genotype coverage of Gardasil 9 protects against some 90% of anal and cervical cancers, as well as large portions of cancers of other anatomic sites.^108^ In summary, while prophylactic HPV vaccines stand to greatly reduce the burden of male HPV diseases and consequent spread to sexual partners, male vaccine eligibility itself lagged behind that of female patients.

Some countries have recognized the public health benefits of prophylactic HPV vaccination and have instituted national government‐funded vaccination programs for adolescents since licensure of the vaccine in their country. Australia, for example, was one of the first countries to institute a national publicly funded school‐based program to vaccinate adolescents, which has resulted in the completion of the prophylactic HPV vaccination series by nearly 80% of Australian girls^109^ and 70% of Australian boys.^110^ With this level of vaccination, Australia has experienced decreases in genital warts and vaccine‐type HPV infections,^111^ as well as herd immunity, in that incidence of genital warts decreased in males prior to initiation of male vaccination.^112^ Comparable effects have been seen in other countries such as Scotland that have implemented similar vaccination programs.^113^ In contrast, countries that have been hesitant to adopt national HPV vaccination policies have not garnered all of the population‐wide benefits reaped by countries with strong vaccination coverage.^111^ The United States is one such country, where poor HPV vaccination rates have limited the public health benefits of prophylactic vaccination. In 2015, 37.1% of females 13 to 15 years and 27.1% of males 13 to 15 years had at least three doses of prophylactic HPV vaccine, a great shortfall from the US Healthy People 2020 goal of 80% vaccine coverage for this age group.^114^ Another study of 2014 to 2015 vaccine uptake data showed that the percentage of 13‐ to 17‐year‐olds in the United States who reported receiving one or more prophylactic HPV vaccine doses increased from 41.7% to 49.8% among males, and from 60.0% to 62.8% among females.^114^ Using 2013 to 2014 NHANES data, Han et al showed an overall vaccination rate among vaccine‐eligible men of only 10.7%.^115^ Using data from the 2015 National Health Interview Survey, 10.1% of men compared with 41.6% of women ages 19 to 26 reported receipt of at least one HPV vaccination dose.^116^ A 2017 Morbidity and Mortality Weekly Report of 2016 data reports 60.4% of 13‐ to 17‐year‐olds of either sex having received at least one vaccine dose, an increase from 56.1% in 2015. Uptake of at least one vaccine dose in adolescent females during this time frame was 65.1% compared with 56.0% in adolescent males.^117^ In summary, US vaccination rates trail those of some developed nations, with men uniformly lagging behind women, although this gap appears to be closing.

Prophylactic HPV vaccination policies for males have been influenced by numerous pharmacoeconomic analyses, reviewed in greater detail elsewhere.^118^, ^119^ Principal determinants of HPV vaccine cost‐effectiveness include vaccination cost per dose, vaccination schedule, gross domestic product per capita, and risk reduction in disease burden among males and females, among others.^118^, ^120^, ^121^, ^122^, ^123^ An additional factor considered in vaccination policy making is the annual medical cost burden of screening for and treating HPV‐related cancer, which has been estimated in the United States at $6.6 billion USD and $1.0 billion USD, respectively, by Chesson and colleagues.^124^ Approximately 30% of HPV‐related cancer treatment costs corresponded to oropharyngeal cancer,^124^ which is known to affect males at higher rates than females. This high cost burden of HPV disease seems to negate the feared expense of extending vaccination indications to include males. Despite this cost burden, earlier analyses concluded that the cost associated with inclusion of males in vaccination programs in the United States exceeded cost‐effective thresholds.^125^ Other studies have shown favorable pharmacoeconomic benefit in the eligibility inclusion of noncervical HPV‐related outcomes, including HR male populations such as MSM.^126^, ^127^, ^128^ In addition to vaccinating HR males as a distinct population, numerous studies have demonstrated population‐level cost‐effectiveness of male vaccination in countries where HPV vaccination rates among women are low.^118^ Although systematic studies must be conducted to confirm this, the recent adoption of a two‐dose regimen (as opposed to the former three‐dose regimen) for boys and girls younger than 15 years old can potentially increase the cost‐effectiveness of prophylactic vaccination in both males and females. In addition, other recent studies have shown promise in a single‐dose vaccination strategy conferring prophylactic benefit.^129^, ^130^ A final economic impediment to vaccine uptake is the cost of vaccination, particularly in the health care environment before the passage of the Patient Protection and Affordable Care Act. A 2009 analysis of online news coverage following the introduction of 4V Gardasil showed approximately half (49.2%) of news coverage referenced concerns about vaccine affordability due to its cost.^131^ Vaccine cost has also been reported to contribute to delayed or low vaccine uptake in low‐ and middle‐income countries.^132^ In MSM, concerns of vaccine cost have been shown to negatively affect intent to become vaccinated.^49^

Other multilevel factors, such as the delayed adoption of sex‐neutral vaccination policies, demographic differences among vaccine‐eligible males, and geographic variation have contributed to low vaccination rates among men. The principal contributing factor to lower vaccination rates among eligible males, particularly in high‐income countries with national HPV vaccination policies, is the delayed adoption of sex‐neutral vaccination policies and publicly funded programs.^133^ For instance, both Australia and the United States adopted male vaccination recommendations many years after the initial recommendation for the vaccination for females. Although not found consistently, socio‐demographic variation exists for prophylactic HPV vaccination coverage among boys and men.^134^ A national study among adolescents in the United States found that HPV vaccination coverage was lowest among non‐Hispanic white boys compared with boys of all other races and ethnicities.^114^ Examination of regional differences found that vaccination coverage was lowest among boys in the southern United States compared with boys in other regions of the United States and was lower among boys living in households at or above the poverty level compared with boys in households living below poverty.^114^ Another study found that boys living in less densely populated nonurban areas had lower odds of initiating and completing the HPV vaccination series compared with boys from more urban areas.^135^ Sexual behavior and self‐reported HIV status are also important factors to consider in the examination of HPV vaccination rates among adult men. Data from the 2014 National HIV Behavioral Surveillance found that 17.2% of MSM ages 18 to 26 years and 37.2% of HIV‐positive MSM ages 18 to 26 had received at least one dose of HPV vaccine, which are higher than rates found among men in the general population.^136^

Newman et al conducted a systematic review and meta‐analysis that identified factors across 23 studies that examined HPV vaccine acceptability among males.^134^ The factors that were most positively correlated were health care professional recommendation and perceived benefits of HPV vaccination. These results were similar to those of a systematic review that reported that the vaccine acceptability for girls was higher in the United States with a belief in vaccine effectiveness, a physician recommendation, and high likeliness of acquiring HPV.^137^ However, weaker and less consistent provider recommendations would likely have contributed to a lower rate of HPV vaccination among males compared with females.^138^ Research has found that despite current more sex‐neutral HPV vaccination recommendations, health care providers provide less consistent and weaker HPV vaccination recommendations to males compared with females.^139^ In European countries at a time when HPV vaccination was only available for girls, parental acceptance of HPV vaccination for boys was as high as that for girls. This was particularly true in countries with active vaccination policies such as the United Kingdom and Italy.^140^ Taken together, dissemination of perceived benefits and strong recommendations by health care providers are two factors grounded in health behavior theory that are closely associated with vaccine uptake by eligible males.

## 4 THERAPEUTIC VACCINATION

In light of the trailing prophylactic HPV vaccination rates in males, many young men remain at risk of not only contracting new infections and succumbing to HPV‐related anogenital or oropharyngeal complications but also to spreading infection to both female and male sexual partners. Additionally, these risks are also present in all men that are older than that targeted age range for prophylactic vaccination. Thus, new therapeutic modalities are needed to address patients with active HPV infection before oncogenic transformation can occur. Standard‐of‐care excisional procedures exist for cervical dysplasia, such as loop electrical excision procedure (LEEP) or cold knife conization.^141^ While these procedures are quite effective at removing dysplastic lesions, LEEP has been shown to double the risk of preterm delivery in future pregnancies.^142^ In male patients, treatment of penile lesions can include a combination of topical (trichloroacetic acid, 5‐fluorouracil, imiquimod, and others), ablative (laser ablation and cryotherapy), and excisional (resection, Mohs micrographic surgery, penectomy, and others) treatments.^143^ Similar treatment modalities may also be employed for the management of AIN lesions.^144^ These procedures, like LEEP, are not without negative side effects that can be disfiguring and/or subsequently alter psychosexual wellness.^145^ Because of these negative side effects of excisional/ablative therapy, numerous HPV therapeutic vaccines have been developed and are at various stages of clinical and preclinical testing, reviewed in great detail elsewhere.^146^

One such therapeutic HPV vaccine currently being tested in male patients is SLP‐HPV‐01, in the Therapeutic HPV‐16 Vaccination for the Treatment of Anal Dysplasia (VACCAIN‐T) study (clinicaltrials.gov identifier: NCT01923116). This vaccine is comprised of nine synthetic HPV 16 E6 peptides and four synthetic HPV 16 E7 peptides and is administered with or without interferon‐α. It is designed to regress HPV 16‐positive AIN 2/3 lesions in HIV‐positive MSMs with recurrent disease or previous unsuccessful treatment, with a follow‐up period of up to 18 months in a phase 1/2 study. Although no data are currently available from this study, the same vaccine has been shown to be efficacious in HPV 16‐positive vulvar intraepithelial neoplasia 3 (VIN3),^147^ HPV 16‐positive cervical HSIL,^148^ HPV 16‐positive gynecologic carcinoma,^149^ and advanced/metastatic cervical cancer.^150^ This same vaccine is also currently being tested (clinicaltrials.gov identifier NCT02426892) in combination with nivolumab in patients of both sexes with HPV 16‐positive incurable solid tumors of the oropharynx, vulva, vagina, anus, or penis. This phase 2 study has a follow‐up of 11 weeks, and neither of these SLP‐HPV‐01 trials have data currently available. Thus, male therapeutic vaccination for HPV‐related complications may become a viable treatment option in the future.

Although Gardasil is perhaps the most common prophylactic HPV vaccine, another trial is currently testing 4V Gardasil as a means of preventing recurrence of high‐grade AIN in MSMs who were previously successfully treated. The HPV Vaccination After Treatment of AIN (VACCAIN‐P) study (clinicaltrials.gov identifier NCT02087384) is comparing 4V Gardasil to normal saline (placebo) in a randomized, double‐blind fashion with a follow‐up of up to 18 months. Secondary outcome measures also include cumulative occurrence of anogenital warts and HPV type‐specific antibody responses. While there are currently no data available from this study, a previous study shows potential for the therapeutic efficacy of 4V Gardasil.^151^ This observational, nonconcurrent cohort study by Swedish and colleagues compared recurrence of high‐grade AIN in HIV‐negative MSMs that were either unvaccinated (n = 114) or had received three vaccinations of 4V Gardasil (n = 88). This study showed that vaccination with Gardasil was associated with decreased risk of histologic recurrence of high‐grade AIN at 2 years after study entry. Although it is not well‐documented whether subjects currently had AIN at the time of vaccination (and, therefore, whether the vaccine is therapeutic per se), this study provides proof‐of‐principle for HPV vaccination in men with therapeutic intent. Similarly, 4V Gardasil has shown promise at preventing recurrence of high‐grade CIN lesions in women post‐LEEP.^152^ Taken together, while most attention has been focused on the development of therapeutic vaccines that stimulate T‐cell responses, prophylactic HPV vaccines are being investigated in a therapeutic manner.

## 5 CONCLUSIONS

HPV infection is, by far, the most common sexually transmitted viral disease. Only a minority of patient infections will progress to produce symptoms such as cervical, penile, or AINs, a minority of which will progress to overt cancer. While men can develop oncogenic HPV infections, women tend to present more commonly with symptomatic disease, which substantiates men as a viral reservoir. Coinfection with HIV, increased numbers of lifetime sexual partners, and earlier age at sexual debut appear to be strongly and independently associated with the development of anogenital HPV infections. PIN, AIN, and associated cancers and treatments can have deleterious effects on psychosexual wellness. Oropharyngeal HPV infections and OSCC cases are on the rise and are predicted to overtake the number of cervical cancer cases in the coming years. This trend is exacerbated by the higher prevalence of oropharyngeal HPV in men, compared with women. Because there are neither treatments for pre‐symptomatic HPV infection nor approved male HPV screening tests, most men remain unaware of HPV infection. These epidemiologic data demonstrate that males are a viral reservoir, a phenomenon that must be addressed to reduce population‐level HPV infections and their sequelae.

One such population‐level mechanism to reduce male HPV infection is prophylactic vaccination. Gardasil 9 confers protection from infection with LR HPV genotypes that are responsible for >90% of all genital warts, and HR HPV genotypes that are responsible for 90% of anal cancers and large proportions of penile and oropharyngeal cancers. Male vaccination serves not only to protect men from HPV‐related disease but also to contribute to herd immunity, thereby reducing HPV disease burden in both sexes. Unfortunately, male vaccination rates have trailed those in women and girls in the United States due to a variety of factors. Delayed adoption of sex‐neutral vaccination policies prevented now vaccine‐eligible males from contributing to herd immunity. Health care providers have been less consistent and less assertive in recommending male patients to become vaccinated. These factors have contributed to a large population of unvaccinated males who may later succumb to disease.

Currently, there is not an approved therapeutic vaccine for HPV infections, although many remain under investigation and development. Therapeutic vaccinations vary from prophylactic vaccinations in both modality and design. Therapeutic vaccinations are under development for individuals with high‐grade AIN and CIN and are designed to stimulate T‐cell immune responses, as opposed to the stimulation of neutralizing antibodies by prophylactic vaccinations. Scant studies have shown therapeutic potential of Gardasil, but larger sample follow‐up studies are needed to confirm this. If therapeutic HPV vaccination trials in men are successful, they will likely play a role in addressing current and future infections in men directly. Men may also derive indirect benefit via their vaccinated female sexual partner(s), if these studies in women are successful.

## 6 FUTURE DIRECTIONS

Addressing the HPV disease burden of men themselves and their sexual partners will require a multifaceted prophylactic approach from both biological and public health perspectives. In order to decrease immediate HPV spread, aggressive prophylactic vaccination programs targeting young men and boys (ideally before sexual debut) must take place. Mandatory HPV vaccination for school enrollment is one avenue to achieve this, although this remains controversial.^153^ School‐based education programs must include HPV‐related disease, spread, and prophylaxis in a comprehensive sex education curriculum, with a special emphasis on prophylactic vaccination and general HPV literacy. At the provider level, strong and consistent provider recommendation for HPV vaccination of boys and men must be used to increase vaccination among eligible patients. Efforts to increase the acceptability of HPV vaccination of males should underscore the benefits of vaccination and include culturally tailored materials for young MSM or young men who intend to have sex with men. Welcoming health care environments may foster destigmatized conversations between men and their health care providers, which in turn may allow men to feel comfortable discussing male‐male sexual activity. Finally, policy changes that reduce financial barriers to HPV vaccination among eligible patients must be addressed in both first‐time and “catch‐up” vaccine recipients. Given the nature of this critical public health issue in both men and their sexual partners, a combination of these strategies is recommended by the Community Preventative Services Task Force and the Advisory Committee on Immunization Practices.^154^, ^155^, ^156^

## CONFLICTS OF INTEREST

The authors have declared that there is no conflict of interest.

## DISCLOSURES

This work was supported in part by National Institutes of Health (National Cancer Institute) Grant R01 CA143130. The supporting funding source had no involvement in study design; collection, analysis, and interpretation of data; writing of this report; or the decision to submit this report for publication.

## AUTHOR CONTRIBUTIONS

Conceptualization: Benjamin J. Lieblong

Funding Acquisition: Mayumi Nakagawa

Project Administration: Mayumi Nakagawa

Visualization: Benjamin J. Lieblong

Writing—Original Draft Preparation: Benjamin J. Lieblong, Brooke E. E. Montgomery, L. Joseph Su, Mayumi Nakagawa

Writing—Review and Editing: Benjamin J. Lieblong

All authors have read and approved the final version of the manuscript. Benjamin J. Lieblong had full access to all of the data in this study and takes complete responsibility for the integrity of the data and the accuracy of the data analysis.
